# Treatment of Teeth during Gestation and Lactation

**Published:** 1877-03

**Authors:** J. Williams


### ARTICLE IV.

### *Treatment of Teeth during Gestation and Lactation.*

BY J. WILLIAMS, D. D. S.

Read before the Ohio State Dental Society.

I desire very briefly to discuss a few propositions that seem to me fundamental to the topic now under consideration.

It is believed by some, and, indeed, so far as I know, the opinion obtains generally, that because, during the periods of gestation and lactation, a new osseous system is in process of formation, growth and development, deriving as it does, every atom entering into the composition of its structure from the blood of the mother, therefore, the quantum of bone making material designed for the nutrition of the mother's bones and teeth, is decreased by just so much as has been abstracted for the building up of the new osseous system, and as a consequence her bones, and especially her teeth, are during these periods, very imperfectly nourished. Those who accept and teach this theory, assume that the teeth, especially during gestation, are left in a starved condition, and almost necessarily fall an easy prey to the vitiated secretions found in the oral cavity.

If this hypothesis be true, we would naturally expect to find every other organ and tissue of the body suffering in a like ratio from like causes. But generally we do not find pregnant women becoming emaciated and hollow eyed, the muscles do not become attenuated and weak, the skin does not, for want of nutrition, disintegrate and fall off in patches, the hair and nails in growth and texture continue the same.

To demonstrate the truth of such a hypothesis would prove either a great blunder on the part of the Creator of all things, or that the *Genus Homo* is not yet as thoroughly *evolved* as the other orders of animals. Run through the whole range of comparative physiology, and no such blundering, disjointed phenomena can be found. The lioness brings forth her whelps without loss or abrasion of tooth structure.

We have the best possible evidence that the Divine institutor of law made ample provision for the reproduction of species without necessarily impoverishing the blood of those set apart and consecrated to the reproductive office, and without in any way instituting pathological conditions.

When the female organization has so far developed as to be capable of the gestative function, natural and physiological processes immediately provide for an excess of bone making material sufficient for a new muscular and a new nervous system, etc.

If the life germ that is produced every month during the period of potential maternity be vitalized, then will this surplus be attracted by chemico-vital force to the vitalized germ, and by it be appropriated in the growth and development of a new organization. If this excess supply be not thus appropriated, another physiological process, called catamenia is instituted, whereby this surplus is periodically expelled from the system, because it is not needed, having been provided for another contingency.

Proof is not wanting to show that in normal conditions the supply of bone making material is sufficient to maintain the integrity of the bones and teeth of the mother, and at the same time build a new system. The Indian mother living in a state of nature does not suffer from disintegration of tooth structure. Thousands of women, even in civilized countries, and who have raised large families, have suffered no more from caries of the teeth than males usually suffer. You can all refer to instances where gestation has been commenced and completed with no more decay of the teeth than during the same length of time in the unimpregnated condition.

If, then, maternity be a physiological condition, in perfect harmony with nature and her laws, if it be, as it is, productive of health, vigor and long life, whence arises the pathological conditions so often met with in civilized countries during the periods of gestation and lactation, especially as regards the teeth?

It is not because the growing fœtus has exhausted the supply of the lime phosphates, for when the mother's teeth suffer from defective calcification, facts show that the child has also been poorly supplied with bone material.

The children born of mothers whose teeth have decayed rapidly during gestation, invariably have poor teeth.

Neither will we find a satisfactory solution to the problem on the supposition that the grains, vegetables and meats ordinarily used as food are deficient in the lime phosphates, for as has been shown, the very best results are often attained when only the ordinary diet, consisting of good bread, made from wheat or rye, oat meal, the various fruits and vegetables, good beef, milk and butter, etc., has been used.

The primary causes, it seems to me, are, the premature age at which gestation or child bearing is commenced—systemic unfitness for the reproductive function from other causes, and mental infelicity.

The important functions of gestation can not be sustained without great damage to every organ and tissue of the body until there is a complete and perfect development of the physical powers, until full growth and maturity have been attained.

Every observing dentist knows that it is usually during the first period of gestation that those fearful ravages upon the teeth so often seen, occur. Especially is this true of very young persons. It is not uncommon in this country, for girls of sixteen to form matrimonial alliances, and at or before seventeen give birth to a child.

Is it strange that in such cases there should be a breaking down, not only of the teeth, but of every organ and function of the body? Until there is a perfect and harmonious development, and healthy condition of all the physical, mental and moral faculties, gestation should not be commenced.

That which, more frequently than any one thing, operates as a primary cause of general and special pathological conditions, and which generally gives efficiencies to secondary causes, for want of a better name, may be termed infelicicous mental states.

Judging from the numerous, procured abortions, that form a part of the unwritten history of humanity, as well as from much positive information on the subject, we infer that child bearing is not always, if indeed generally, a matter of choice, but is regarded, very often, as the most distressing calamity that could have occurred. In many instances so great is the mental anguish, that death itself would have been chosen rather than pregnancy, and life and health are often voluntarily placed in jeopardy, in order to be freed from a condition so repugnant. Hope and joy flee away and leave the mind in a state of utter wretchedness. With the mind, the grand propellor of all the physical functions in such a condition, not a single vital function is, nor can be well performed, I care not how rich the food may be in the lime salts, and how much of the bone making material may have been superadded with the nervous and mental stimuli thus withheld, there can be no healthy and complete appropriation of them.

However good the former hygienic regimen may have been, it is suddenly abandoned. However much the teeth may have been valued and cared for, they now receive little or no attention, and their value dwindles into insignificance.

The case is far different with her whose physical and mental powers have attained their full and harmonious development, and in whose breast maternal feelings and desires have been quickened into vigorous life. With the intelligent women thus previously prepared in body, will and affections, and with whom gestation is no accident, and is not regarded as a calamity, digestion, assimilation, oxydation and appropriation will be well performed.

There will be no abandoning of the former excellent hygienic regimen, on the contrary a more careful looking to all its minutia. There is no breaking down of will power, no gloomy foreboding of death and the grave, but a determination to live and give life.

It is scarcely necessary to say that the teeth of such a person will pass the ordeal of gestation and lactation with no more injury than during an equal period of time in the now gestative state.

Notwithstanding gestation is a healthy physiological process, it is a very different condition from the unimpregnated state. There is usually exquisite nervous and mental susceptibility, requiring much knowledge, prudence, skill and delicacy on the part of the physician and dentist. What they do, or neglect to do may greatly effect both mother and child.

What would be eminently proper and advisable in one condition, might be very improper and injurious in the other.

It may, I think, be laid down as a rule, that operations, requiring much time, severe or unpleasant mental impressions, or that may occasion unpleasant associations, should, if possible, be postponed till some considerable time after parturition.

The treatment, so far as the filling of teeth is concerned, should be temporary. No attempt at great thoroughness should be made. Cavities that are superficial should not be disturbed, and those in which decay has so far progressed as to render it probable that exposure of the pulp may take place soon, or in which thermal changes effect the nerve, should be filled with gutta-percha, or oxy-chloride of zinc, and no more excavation should be made than is absolutely necessary.

The capping of the pulps of teeth during gestation, owing to extremely nervous susceptibility, is not as likely to be successful as at other times, but in very important teeth it is always proper to attempt it, rather than resort to devitalization.

I would never extract a tooth during gestation that can be rendered tolerably comfortable by palliative treatment, and in most cases this can be done.

Were it neccessary many instances might be cited to show the impropriety of surgical operations during pregnancy, not imperatively required. Abortions and malformations, as well as mental impressions and influences, lasting during the life of mother and child, are among the sequences.

The agents necessary to be used in correcting abnormal conditions of the fluids of the mouth, can only be determined by testing those fluids. If they are found to be acid, alkaline washes may be prescribed; if found to be alkaline, acidulated washes, and acid fruits are indicated.

Too much importance can not be attached to the regular, frequent and thorough cleansing of the mouth and teeth.—*Dental Register.*

